# Procoagulant Effect of FIX Concentrates and Bypass Agents in Combination with Emicizumab and Impact of FVIII Inhibitors

**DOI:** 10.3390/biomedicines14040777

**Published:** 2026-03-29

**Authors:** Elena G. Arias-Salgado, María Teresa Álvarez Román, Abel Dos Santos Ortas, Ihosvany Fernandéz-Bello, Elena Monzón Manzano, Paula Acuña, Mónica Martín Salces, Maria Isabel Rivas Pollmar, Sara García Barcenilla, Nora V. Butta, Víctor Jimenéz-Yuste

**Affiliations:** 1 Servicio de Hematología y Hemoterapia, Hospital Universitario La Paz, IdiPAZ, Paseo de la Castellana 261, 28046 Madrid, Spain; 2 Facultad de Medicina, Universidad Autónoma de Madrid, Arzobispo Morcillo 4, 28029 Madrid, Spain; 3 Centro de Transfusión de la Comunidad de Madrid, Avenida de la Democracia sn, 28032 Madrid, Spain; 4 Departamento de Hematología y Hemoterapia, Hospital General Universitario Dr. Balmis, 03010 Alicante, Spain

**Keywords:** hemophilia, inhibitors, emicizumab, recombinant factor IX, bypassing agents, global coagulation assays, thrombin generation

## Abstract

**Background/Objectives:** Patients with severe hemophilia A on prophylaxis with emicizumab exhibit a mild/moderate bleeding phenotype that requires the use of either recombinant FVIII (rFVIII) or bypassing agents (BPAs) in patients with inhibitors, in the case of breakthrough bleeding or surgery. Since factor IX (FIX) limits the formation of the FIXa–emicizumab–FX complex, exogenously added FIX might enhance complex formation and thrombin generation. This study aimed to compare the procoagulant effects of various FIX concentrates with recombinant activated FVII (rFVIIa), activated prothrombin complex concentrate (aPCC), and rFVIII in SHA patients with and without inhibitors under emicizumab prophylaxis. **Methods:** Hemostatic changes were monitored using two optimized global coagulation assays: rotational thromboelastometry and calibrated automated thrombin generation. Tubes containing corn trypsin inhibitor (CTI) were used during blood collection to prevent activation. Low concentrations of tissue factor (TF) were used to trigger coagulation in both assays. **Results:** Ex vivo addition of recombinant FIX concentrates significantly increased the procoagulant activity of emicizumab, achieving levels comparable to therapeutic doses of rFVIIa or rFVIII, and the proportion of active FIXa within the concentrates is a major contributor to their procoagulant function. We assessed the influence of FVIII inhibitors on the hemostatic efficacy of rFIX concentrates and BPAs, finding that rFIX-induced thrombin generation increased in the presence of inhibitors, and no significant differences were observed with BPAs. **Conclusions:** These findings suggest that FIX concentrates could be an effective alternative to BPAs for emicizumab-treated patients, particularly those with inhibitors. Further studies are needed to confirm their in vivo efficacy and to evaluate thrombotic risk.

## 1. Introduction

Emicizumab is a humanized, monoclonal, bispecific antibody that recognizes factor (F) FX and FIXa, allowing for thrombin generation in the absence of FVIII [1]. It is used as a prophylactic treatment to prevent bleeding episodes in patients with severe hemophilia A (SHA) with and without inhibitors, as well as in patients with moderate or mild hemophilia A, especially those with a severe bleeding phenotype, and in patients with difficulty with venous access [2].

When administered at therapeutic levels (approximately 50 µg/mL), emicizumab exhibits an FVIII-equivalent activity of 10–15%, as estimated by various assays [3,4]. Indeed, the bleeding rates observed in SHA patients undergoing prophylaxis with emicizumab are comparable to those observed in patients with mild HA. Nevertheless, some patients on emicizumab prophylaxis still require FVIII concentrates (for those without inhibitors) or BPAs (for those with inhibitors) in the event of acute bleeding or surgical procedures [5,6]. In patients with inhibitors, this leads to the administration of recombinant activated FVII (rFVIIa) or activated prothrombin complex concentrate (aPCC), whose efficacy remains suboptimal and are not free of thrombotic risks [5,7,8,9,10,11,12,13].

The monitoring of the hemostatic potential of emicizumab in the presence or absence of BPAs or FVIII concentrates has been optimized. Several global coagulation assays have proven useful in this context, including rotational thromboelastometry (ROTEM^®^) [14,15], clot waveform analysis (CWA) [15,16], and the thrombin generation test (TGT) [7,17]. An important consideration is the trigger reagent used in these assays. Several studies suggest that assay conditions employing low concentrations of tissue factor (TF), which more closely mimic physiological coagulation, are more effective in distinguishing treatment responses and bleeding phenotypes [18].

Under normal physiological conditions, plasma concentrations of FIX and FX are approximately 90 nM and 135 nM, respectively, while the therapeutic concentration of emicizumab is around 370 nM. For that reason, in SHA patients on emicizumab prophylaxis, thrombin generation is primarily dependent on FIX levels, which limits the formation of the FIXa–emicizumab–FX ternary complex [1]. The in vitro ability of FIX to enhance emicizumab-dependent thrombin generation has been previously reported [7]. Therefore, it is reasonable to hypothesize that increasing FIX concentrations might improve emicizumab procoagulant function. Unlike rFVIIa or aPCC, FIX replacement therapies are typically administered in their zymogen (inactive) form and, importantly, their efficacy can be monitored using standard assays for plasma FIX activity, whereas no standardized methods exist to assess the procoagulant activity exerted by rFVIIa or aPCC [19].

In this context, we aimed to use two global coagulation assays to compare the ex vivo procoagulant effect of therapeutic concentrations of BPAs and various recombinant FIX (rFIX) concentrates in samples from SHA patients on emicizumab prophylaxis.

Furthermore, the impact of FVIII inhibitors on the hemostatic response of emicizumab remains to be investigated. To elucidate the influence of the presence of FVIII inhibitors, we aimed to analyze possible differences in the procoagulant effects of BPAs and rFIX products in samples from emicizumab-treated patients comparing those with and without FVIII inhibitors.

## 2. Materials and Methods

### 2.1. Subjects

Twenty patients with SHA, 45 [27–54] years old (median [IQR]), 40% (8) with inhibitors, on emicizumab prophylaxis for >18 months, were recruited at the Hospital Universitario La Paz in a single-visit study. Patients were consecutively included according to predefined inclusion and exclusion criteria, which ensured that they had severe hemophilia A, were on stable emicizumab prophylaxis, and had no conditions or treatments that could interfere with the study. Patients who had received rFVIIa, aPCC, or FVIII concentrates within 24, 48, or 72 h prior to sampling, respectively, were excluded.

Sixteen healthy male subjects, 40 [29–47] years old, formed the control group. Healthy controls were defined as age- and sex-matched subjects without known hemostatic disorders, relevant medical conditions, or medications affecting coagulation.

The prophylactic dose of emicizumab was 1.5 mg/kg/week in 55% (11), 3 mg/kg/every two weeks in 10% (2), or 6 mg/kg/every four weeks in 35% (7) of the patients.

### 2.2. Samples

Blood samples were obtained by venipuncture using BD Vacutainer Safety-Lok™ (BD Vacutainer, Madrid, Spain) blood collection sets with a 21-gauge needle. Samples were collected using tubes with 3.2% sodium citrate (BD Vacutainer, Madrid, Spain) and tubes with citrate and corn trypsin inhibitor (CTI) (SCAT type27, CellSystems, Troisdorf, Germany). The use of CTI prevents the contact pathway activation and reduces variability from sample collection [20]. Platelet-poor plasma (PPP) was obtained using a PDQ Platelet Function Centrifuge (Bio/Data Corporation, Horsham, PA, USA). Aliquots were stored at −80 °C until analysis.

Samples from SHA patients were spiked with different concentrations of a recombinant (r) rFVIII (octocog-alfa, Advate^®^, Baxter), BPAs [rFVIIa (eptacog alfa activated, NovoSeven^®^, NovoNordisk), aPCC (activated prothrombin complex concentrate, FEIBA, Takeda)], or different recombinant FIX concentrates: two standard-half-life (SHL) rFIX [rFIX-alfa (nonacog-alfa, BeneFIX^®^, Pfizer) and rFIX-gamma (nonacog-gamma, Rixubis^®^, Takeda)], and one extended-half-life (EHL) fusion protein with albumin [rFIX-FP (albutrepenonacog-alfa, Idelvion^®^, CSL Bering)]. Octocog-alfa and eptacog-alfa were selected as representative recombinant hemostatic agents, widely used in clinical practice and commonly employed as reference comparators in experimental coagulation studies.

Samples from healthy controls were used to establish a reference range for the global coagulation assays under our experimental conditions. Blood collection and laboratory assays for controls were performed on the same day and processed under identical experimental conditions as patient samples.

A diluted one-stage FVIII assay on the ATELLICA^®^ analyzer (Siemens, Madrid, Spain) employing an emicizumab calibrator (r^2^ diagnostics, Stago, Spain) was used to measure the emicizumab levels in plasma samples [21].

### 2.3. Rotational Thromboelastometry (ROTEM^®^)

Clot formation was assessed by rotational thromboelastometry using a ROTEM^®^ Delta (Werfen, Madrid, Spain), as previously described [22]. Blood obtained in citrate was evaluated with the NATEM test triggered by recalcification only (Start-TEM), and blood collected with CTI was activated with a low concentration of tissue factor (TF) by diluting the EXTEM reagent 50,000-fold in HEPES solution (20 mM HEPES, 150 mM NaCl, 2% bovine serum albumin, pH 7.4). This dilution corresponds to approximately 0.1 pM TF, as estimated by a TF/FVIIa-mediated FX activation assay [23]. ROTEM^®^ reagents were from Werfen, Madrid, Spain.

Clotting time (CT), clot formation time (CFT), amplitude at 5 min (A5), and time to the maximum velocity of clot formation (MAXV-t) were evaluated.

### 2.4. Calibrated Automated Thrombography (CAT)

Thrombin generation was assessed as previously described [22] in PPP obtained from blood with CTI by calibrated automated thrombography (CAT). Coagulation was initiated by recalcification (FluCa kit, Diagnostica Stago, Madrid, Spain) and the addition (final concentrations) of 1 pmol/L of recombinant human TF and 4 µmol/L of phospholipid mixture (PPP-Reagent LOW, Diagnostica Stago, Madrid, Spain) to the sample. Thrombin was measured using a Fluoroskan FL microplate reader (Thermo Scientific, Madrid, Spain) with the Thrombinoscope software, v3.6 (Thrombinoscope BV, Maastricht, Holand).

Lag time (LT), peak height (Peak), endogenous thrombin potential (ETP), and time-to-peak (ttPeak) were evaluated.

### 2.5. Treatment of FIX Products with EGRck

rFIX concentrates were incubated with EGRck (Glu-Gly-Arg-chloromethylketone) (CellSystems, Troisdorf, Germany) to irreversibly inhibit activated FIXa fraction. FIX products were incubated 1 h at 37 °C with a 5-fold molar excess of EGRck. Unbound EGRck was removed by extensive dialysis using a Slide-*A*-Lyzer^TM^ MINI-Dialysis Device (3.5K MWCO) (Thermo Scientific, Madrid, Spain). rFIX concentrates not treated with EGRck were also incubated and dialyzed under similar conditions.

### 2.6. Analysis of FIXa Activity

FIXa activity was quantified using a chromogenic substrate for factor IXa (Spectrozyme^®^ FIXa substrate, LOXO GmbH, Dossenheim, Germany), according to the manufacturer’s instructions. Briefly, 20 µL of FIX products diluted in 200 µL of a reaction buffer (50 mM Tris, pH 7.4, 100 mM NaCl, 5 mM CaCl_2_, 40% (vol/vol) ethylene glycol) was added to 96-well microplates and substrate cleavage was initiated by the addition of 25 µL of Spectrozyme^®^ FIXa (10 mM). The chromogenic substrate was analyzed by monitoring the optical density (OD) at 405 nm. Percentage of activated FIXa was calculated from the values obtained with purified activated human hFIXa (HCIXA-0050, CellSystems, Troisdorf, Germany) added as a standard reference in the same assay.

### 2.7. Development of FVIII Inhibitor in Hemophilia a Mice

To generate neutralizing antibodies against human FVIII, hemophilia A knock-out mice (B6;129S-F8-tm1 Kcz/J), 8–10 weeks-old, were treated with 6 intravenous tail injections of human rFVIII (Kovaltry^®^, Bayer, 80 IU/kg), once every two weeks. Two weeks after the last injection, blood from treated mice was collected from the vena cava in 0.1 volume of 3.2% sodium citrate, and platelet free plasma (PFP) was obtained by centrifugation. The titer of inhibitory antibodies against human FVIII was measured in mouse plasma using the standard Nijmegen–Bethesda assay. All treated mice developed high titers (>30 BU) of anti-hFVIII inhibitor antibodies.

### 2.8. Statistical Analysis

Data were analyzed following standard criteria and GraphPad Prism version 6 (GraphPad Software, San Diego, CA, USA) was used for statistical analysis. The Shapiro–Wilk test was used to test normal distribution. Variables were expressed as mean and standard deviation (SD) or median and interquartile range (IQR), as appropriate. Comparison between two groups was carried out either using the unpaired 2-tailed Student’s *t*-test or Mann–Whitney’s *t*-test, as appropriate. For multiple comparisons, a one-way ANOVA followed by Tukey’s post hoc test was used for normally distributed data, whereas a Kruskal–Wallis test followed by Dunn’s post hoc test was used for non-normally distributed data. Correlation was evaluated with Pearson or Spearman tests, depending on the sample distribution. Statistical significance was set as *p* < 0.05.

## 3. Results

### 3.1. Patients’ Characteristics

Twenty patients with SHA on emicizumab prophylaxis, eight with inhibitors, were recruited to participate in this study. Clinical characteristics, treatments, complete blood count, coagulation parameters, bleeding rates, and emicizumab levels are reported in Table 1 and Supplementary Table S1.

SHA patients with inhibitors were younger than patients without inhibitors (median [IQR] years SHA_Inh: 24 [17–39]; SHA_woInh: 52 [44–54]; *p* < 0.02); erythrocyte count was slightly higher in patients with the inhibitor, but both were within the normal range (median [IQR] RBC count (×10^6^/µL) SHA_Inh: 5.4 [5.1–5.6]; SHA_woInh: 4.9 [4.8–5.4]; *p* < 0.05) (Table 1); and most of the SHA patients with inhibitors (62.5%) were not receiving concomitant medication (Supplementary Table S1).

### 3.2. Ex Vivo Analysis of the Procoagulant Effect of BPAs or rFVIII in Emicizumab-Treated SHA Patients Assayed with Global Coagulation Tests

To validate the optimal conditions for monitoring the procoagulant profile of emicizumab-treated patients, two different ROTEM^®^ tests were initially evaluated. When the NATEM test was performed with the citrated blood sample, the ROTEM^®^ parameters observed in the emicizumab-treated SHA patients were similar to the values obtained in healthy controls (Supplementary Figure S1). In contrast, when ROTEM^®^ was performed using blood collected with CTI and activated by a low concentration of TF (final 1:50,000 dilution of EXTEM reagent), CT, CFT, and MAXV-t values were significantly prolonged (*p* < 0.01) in emicizumab-treated patients compared to the controls (Supplementary Figure S1). Data obtained under these ROTEM^®^ conditions demonstrate that emicizumab prophylaxis did not fully restore coagulation parameters in the SHA patients. Consequently, these conditions were selected for the subsequent experiments.

**Figure 1:**
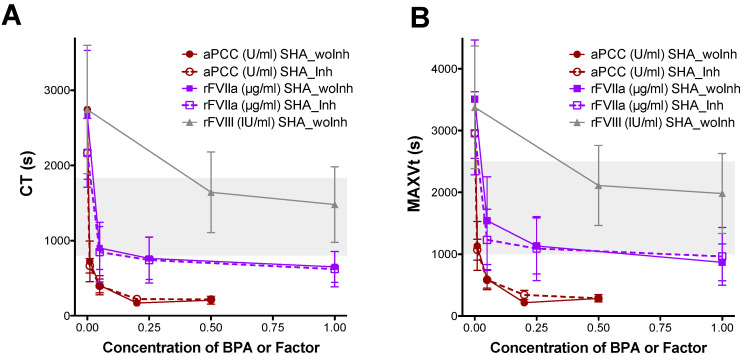
Evaluation of clot formation in blood-CTI samples from emicizumab-treated SHA patients, with and without FVIII inhibitor, after ex vivo addition of bypassing agents or rFVIII. (**A**) Clotting time (CT) and (**B**) time to maximum velocity of clot formation (MAXVt) obtained by ROTEM^®^, before and after the ex vivo addition of aPCC (0.01, 0.05, 0.2 and 0.5 U/mL), rFVIIa (0.05, 0.25, and 1 µg/mL), or rFVIII (0.5 and 1 IU/mL) in blood collected with CTI from the emicizumab-treated SHA patients, with (SHA_Inh) and without inhibitor (SHA_woInh), using EXTEM reagent diluted 1:50,000. Dots and error bars represent the mean ± SD. Grey-shaded areas correspond to the reference range obtained from healthy controls.

The ex vivo procoagulant effect of different therapeutic concentrations of BPAs and rFVIII was evaluated by ROTEM^®^ using samples from emicizumab-treated patients, with and without inhibitors. Addition of 0.01 U/mL of aPCC (equivalent to plasma levels achieved after a dose of 0.5 U/kg) reduced the CT (Figure 1A) and MAXVt (Figure 1B) values to within the control reference range. Increasing concentrations of 0.2 and 0.5 U/mL aPCC (equivalent to 10 and 25 U/kg, respectively) resulted in the shortening of CT exceeding the normal reference range. The addition of 0.25 µg/mL rFVIIa, (equivalent to 25 μg/kg) also normalized the ROTEM^®^ parameters and a saturated response was observed at higher concentrations (1 µg/mL rFVIIa). Addition of rFVIII in samples of patients without inhibitors produced the shortening of CT and MAXVt values normalizing the hemostatic profile within the normal physiological range in all of the assayed concentrations (0.5 and 1 IU/mL). Emicizumab-treated SHA patients with and without the inhibitor did not show any significant differences in ROTEM^®^ variables after the spike addition of aPCC or rFVIIa (Figure 1) (see Supplementary Table S3 for the statistical analysis results).

**Figure 2:**
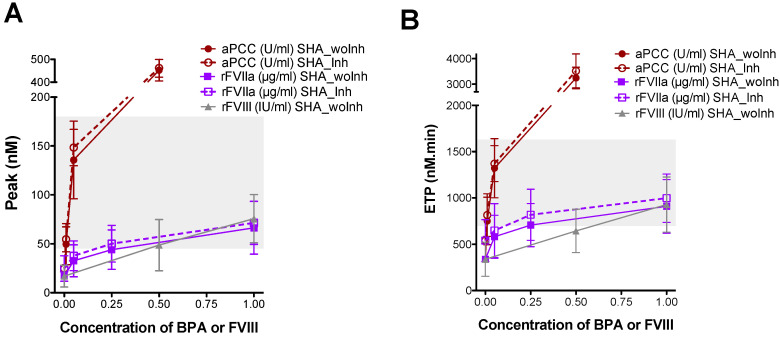
Evaluation of thrombin generation in CTI plasma of emicizumab-treated SHA patients with and without FVIII inhibitor, after ex vivo addition of bypassing agents or rFVIII. (**A**) Peak (maximum concentration of thrombin, nM) and (**B**) ETP (endogenous thrombin potential, nM.min) obtained by CAT, before and after the ex vivo addition of aPCC (0.01, 0.05, and 0.5 U/mL), rFVIIa (0.05, 0.25, and 1 µg/mL), or rFVIII (0.5 and 1 IU/mL) to plasma collected with CTI from emicizumab-treated SHA patients, with (SHA_Inh) and without inhibitor (SHA_woInh). The reaction was triggered with 1 pM tissue factor and 4 μM phospholipids (PPP-low reagent, Stago). Dots and error bars represent the mean ± SD. Grey-shaded areas correspond to the reference range obtained from healthy controls.

The thrombin generation (TG) profile in CTI plasma from emicizumab-treated SHA patients and the ex vivo effect of the addition of BPAs or rFVIII on TG parameters were evaluated by CAT using low levels of TF (1 pM) to trigger coagulation. The baseline levels of the thrombin peak (Figure 2A) and ETP (Figure 2B) were lower in all emicizumab-treated SHA patients compared to the healthy controls, indicating that prophylaxis with emicizumab was not able to completely restore the capacity of thrombin generation in the plasma of the SHA patients.

The addition of 0.05 U/mL of aPCC (equivalent to 2.5 U/kg) restored the TG parameters to within the normal range (Figure 2), whereas 0.5 U/mL aPCC (equivalent to 25 U/kg) resulted in TG parameters levels above the normal reference range, showing a dose-dependent response. With the addition of 0.25 µg/mL rFVIIa or 0.5 IU/mL rFVIII, the thrombin peak (Figure 2A) and ETP (Figure 2B) values reached normal levels, but a saturated response was observed at higher concentrations (1 µg/mL rFVIIa or 1 IU/mL rFVIII). Emicizumab-treated patients, both with and without inhibitors, showed similarly reduced thrombin generation levels, and spiking of plasma from SHA patients with aPCC or rFVIIa produced a comparable procoagulant effect in both groups (Figure 2) (see Supplementary Table S4 for statistical analysis results).

### 3.3. Ex Vivo Analysis of the Procoagulant Effect of Several FIX Concentrates in Emicizumab-Treated SHA Patients Assayed with Global Coagulation Tests

The hemostatic effects of various rFIX concentrates in samples from emicizumab-treated SHA patients were evaluated using the ROTEM^®^ and CAT assays, under the same experimental conditions previously established for BPAs and rFVIII. Three different rFIX concentrates were tested: two SHL-rFIX, rFIX-alfa (nonacog-alfa, BeneFIX^®^) and rFIX-gamma (nonacog-gamma, Rixubis^®^), and one EHL-fusion protein with albumin, rFIX-FP (albutrepenonacog-alfa, Idelvion^®^).

**Figure 3:**
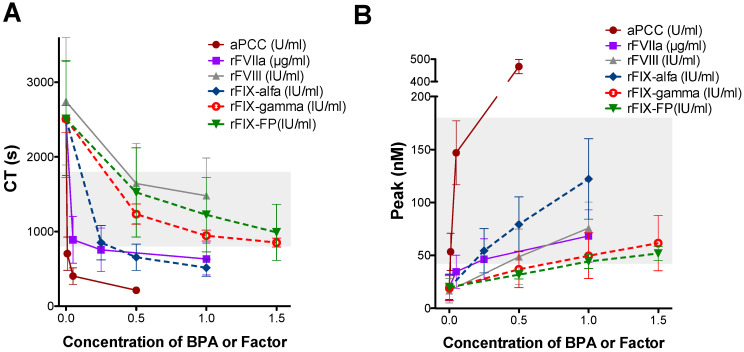
Ex vivo analysis of the procoagulant effect of different rFIX concentrates in emicizumab-treated SHA patients. (**A**) Clotting time (CT) and (**B**) thrombin peak obtained by the ROTEM^®^ and CAT assays after the addition of increasing concentrations of rFIX-alfa (0.25, 0.5, and 1 IU/mL), rFIX-gamma (0.5, 1, and 1.5 IU/mL), rFIX-FP (0.5, 1, and 1.5 IU/mL), aPCC (0.01, 0.05, and 0.5 U/mL), rFVIIa (0.05, 0.25, and 1 µg/mL), or rFVIII (0.5 and 1 IU/mL) to samples collected with CTI from emicizumab-treated SHA patients. Dots and error bars represent the mean ± SD. Grey-shaded areas correspond to the reference range obtained from healthy controls.

The concentrations used for these assays were selected to represent clinically relevant plasma levels achievable after standard therapeutic dosing (for instance, 0.5 IU/mL of rFIX-alfa approximates the expected peak plasma concentration after a standard dose of ~50 IU/kg). Ex vivo spiking of increasing concentrations of all rFIX products showed a dose-dependent shortening of CT (Figure 3A) and an increase in thrombin peak (Figure 3B). The addition of 1 IU/mL of either rFIX-gamma or rFIX-FP, or a lower concentration of rFIX-alfa (0.25 IU/mL), restored the CT (Figure 3A) and thrombin peak (Figure 3B) to the range observed in healthy subjects. These values were similar to those obtained with 0.25 µg/mL of rFVIIa (equivalent to ~25 μg/kg), 0.05 U/mL of aPCC (equivalent to ~2.5 U/kg), or 1 IU/mL of rFVIII in patients without inhibitors. It is important to note that these ex vivo findings do not imply clinical dose interchangeability. The statistical results of the comparisons between the different products and concentrations tested are summarized in Supplementary Tables S5 and S6.

To assess whether the observed difference in the procoagulant activity of rFIX-alfa was due to its higher content of FIXa, we quantified the levels of FIXa in all FIX products tested as described in Section 2.

We observed that rFIX-alfa exhibited six-times higher FIXa levels than rFIX-FP and four-times higher than rFIX-gamma (Table 2), in agreement with previous studies [24,25]. Comparable FIXa activity was observed between the rFIX-gamma and rFIX-FP products.

To confirm the contribution of FIXa to the hemostatic effect of the rFIX concentrates, all products were incubated with the tripeptide EGR-chloromethylketone (EGRck) to irreversibly inhibit and block active FIXa. After treatment with EGRck, we verified that all recombinant products showed an almost complete absence of FIX activity (Table 2).

**Figure 4:**
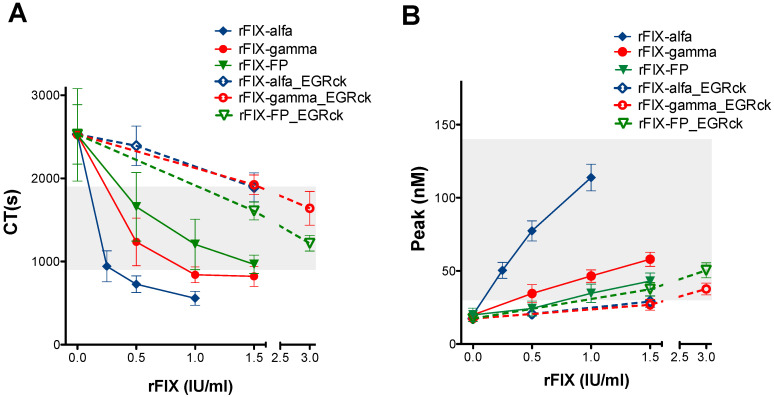
Contribution of the active form FIXa to the procoagulant effect of rFIX concentrates in emicizumab-treated SHA patients. (**A**) Clotting time (CT) and (**B**) thrombin peak obtained by the ROTEM^®^ and CAT assays respectively, after the addition of increasing concentrations of untreated (filled symbols) or EGRck-blocked (open symbols) rFIX concentrates (rFIX-alfa, rFIX-gamma, and rFIX-FP) to samples collected with CTI from emicizumab-treated SHA patient. Dots and error bars represent the mean ± SD. Grey-shaded areas correspond to the reference range obtained from healthy controls.

All the EGRck-treated rFIX concentrates exhibited similar CT values in the ROTEM^®^ assay (Figure 4A) and thrombin peak values in the CAT assay (Figure 4B). The procoagulant effects of the EGRck-treated rFIX concentrates were reduced compared to the effects observed in their untreated counterparts. Differences in CT (Figure 4A) and thrombin peak (Figure 4B) between EGRck-treated and untreated rFIX products were more pronounced for rFIX-alfa (fold change in CT: 3.29; fold change in peak: 3.85), the concentrate with the highest FIXa content (>6%). In contrast, only minor differences were observed between EGRck-treated or untreated rFIX-FP (fold change in CT: 1.61; fold change in peak: 1.13), which contained very low levels of FIXa (≤1%). Supplementary Table S7 shows the statistical differences between the CT and peak values obtained for the untreated and EGRck-blocked rFIX concentrates.

### 3.4. Evaluation of Differences in the Procoagulant Effect of FIX Concentrates Between Emicizumab-Treated Patients with and Without Inhibitors

**Figure 5:**
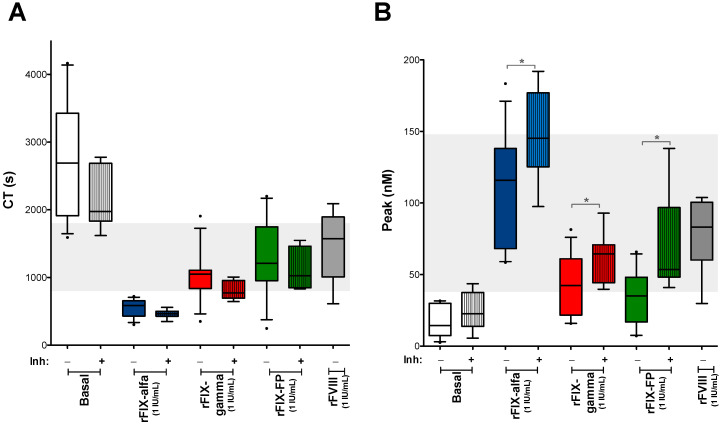
Comparison of the procoagulant effect of rFIX concentrates between emicizumab-treated SHA patients with and without inhibitors. (**A**) Clotting time and (**B**) thrombin peak obtained by the ROTEM^®^ and CAT assays respectively, after the ex vivo addition of rFIX concentrates (rFIX-alfa, rFIX-gamma, and rFIX-FP) (1 IU/mL for all the rFIX products assayed) or rFVIII (1 IU/mL) to CTI samples from emicizumab-treated SHA patient with (+) or without inhibitors (−). Box plots show the 90/10 percentile at the whiskers, with any points outside this range plotted as individual dots, and the median shown as a line. Grey-shaded areas correspond to the reference range obtained from healthy controls. * *p* < 0.05.

The reduction in CT observed by ROTEM^®^ after spiking with rFIX was similar in samples from the emicizumab-treated patients, whether they had inhibitors or not (Figure 5A). However, in the CAT assays, all tested rFIX concentrates resulted in a significantly higher increase in thrombin generation in samples from patients with inhibitors (Figure 5B).

**Figure 6:**
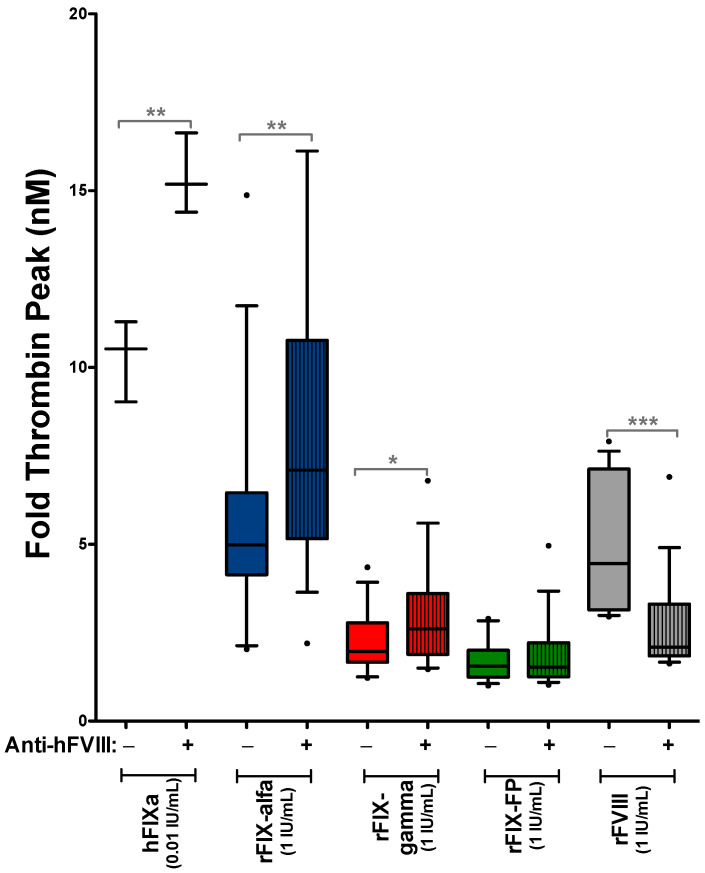
Influence of neutralizing anti-hFVIII antibodies on the procoagulant effect of rFIX concentrates in samples from emicizumab-treated SHA patients. Plasma from emicizumab-treated SHA patients without inhibitors was pre-diluted (dilution 1:30) with HA-mice plasma, either with (+) or without (−) neutralizing anti-hFVIII titers. The figure shows the thrombin peak changes (fold) induced by the ex vivo addition of different rFIX concentrates (rFIX-alfa, rFIX-gamma, and rFIX-FP) (1 IU/mL for all the rFIX products assayed) or purified active hFIXa (0.01 IU/mL). As a positive control of the inhibitory capacity of the mouse neutralizing anti-hFVIII antibodies, we also spiked the samples with rFVIII (1 IU/mL). Box plots show the 90/10 percentile at the whiskers, with any points outside this range plotted as individual dots, and the median shown as a line. * *p* < 0.05; ** *p* < 0.01; *** *p* < 0.001.

To confirm that the greater increase in thrombin peak observed in patients with inhibitors following the addition of rFIX concentrates was indeed due to the presence of neutralizing anti-FVIII antibodies and not attributable to other potential differences between patient groups (such as age, F8 gene variant or concomitant medication), we analyzed the thrombin generation using only samples from patients without inhibitors. We then compared the procoagulant effect of rFIX products and purified hFIXa in the absence or presence of mouse-derived neutralizing anti-hFVIII antibodies during the assay. The inhibitory capacity of anti-hFVIII antibodies was confirmed by their ability to reduce rFVIII-induced thrombin generation (Figure 6). These assays showed that the increase in thrombin peak induced by rFIX, or purified hFIXa, was significantly greater in the presence of neutralizing anti-hFVIII (Figure 6).

Genetic analysis was available for 17 patients (Supplementary Table S2) to evaluate potential differences associated with F8 gene variants. The most frequent alteration was intron 22 inversion (Int22Inv), identified in 7 patients (41%), consistent with the most prevalent genetic abnormality reported in SHA. To assess whether the higher thrombin peak observed in patients with inhibitors could be influenced by F8 mutation type, thrombin generation was compared after the addition of rFIX concentrates (1 IU/mL) in the subgroup of patients carrying the same variant (Int22Inv): 5 patients with inhibitors and 2 without inhibitors. Despite the limited sample size, patients with inhibitors exhibited significantly higher thrombin peaks (Supplementary Figure S2) using Welch’s correction for statistical analysis.

## 4. Discussion

Emicizumab represents a major advancement in the treatment of HA, introducing a novel mechanism of action, a bispecific monoclonal antibody that mimics the cofactor function of activated FVIII by bridging FIXa and FX. Despite this innovation, emicizumab provides only a partial correction of hemostasis, equivalent to approximately 10–15% of normal FVIII activity [3,4]. Consequently, emicizumab-treated patients, particularly those with inhibitors, still require additional hemostatic agents such as BPAs or FVIII (in patients without inhibitor) during breakthrough bleeding episodes or surgical procedures.

Accurate monitoring of coagulation status in emicizumab-treated patients remains challenging because emicizumab does not need to be activated by thrombin, leading to a more pronounced shortening of aPTT than that observed with FVIII [26]. Consequently, aPTT-based assays are inadequate for monitoring emicizumab activity or assessing a patient’s hemostatic potential. In this context, global coagulation assays have been proposed as more appropriate methods [7,8,14,15,27,28]. Their main advantage is the ability to comprehensively evaluate clot formation using whole blood or to monitor thrombin generation kinetics, providing a more physiologically relevant measure of hemostasis. These methods may offer a practical approach to individualize perioperative or on-demand treatment strategies in emicizumab-treated patients. However, these assays were not originally optimized or standardized to assess the effects of bispecific antibody therapies. Our study showed that when performed under optimized conditions, the ROTEM^®^ and CAT results are representative of the in vivo hemostatic effect of emicizumab prophylaxis, and these assays can monitor the ex vivo effects of concomitant hemostatic agents. A crucial aspect was the use of CTI during blood collection to inhibit contact pathway activation, thereby minimizing in vitro artifacts [3,20] and ensuring that coagulation is mainly initiated via the TF pathway, closely mimicking the physiological conditions. Several studies have employed various activators, such as ellagic acid, FXIa, or TF [3,17,29]. While FXIa can be useful for examining FIX activation specifically, it bypasses important upstream and downstream interactions, limiting its ability to capture the full complexity of the coagulation response. In the context of emicizumab, our data indicate that low concentrations of TF are the most informative trigger when the contact phase is blocked by CTI. We successfully used 1 pM TF and a 1:50,000 dilution of the EXTEM reagent (corresponding to approximately 0.1 pM TF as according to a TF/FVIIa-mediated FX activation assay) [23] as triggers of the CAT and ROTEM^®^ assays respectively. This experimental ROTEM^®^ condition was useful for detecting the suboptimal hemostasis in emicizumab-treated patients compared to healthy controls. The ROTEM^®^ assay using the NATEM test on samples collected without CTI, although previously used by other authors [14,15], proved to be less sensitive in our study, showing completely normalized hemostatic parameters with emicizumab (Supplementary Figure S1).

Emicizumab therapy has several advantages, however, in certain clinical contexts such as trauma, surgery, or severe bleeding, additional hemostatic treatments, including BPAs or FVIII, are still required. In patients without inhibitors, numerous studies have demonstrated the efficacy and safety of concomitant use of emicizumab and FVIII, providing reliable hemostatic coverage [22,30,31]. This enhanced effectiveness is primarily attributed to the higher binding affinities of FVIII for FIXa and FX (in nanomolar range) compared to the lower affinities (in micromolar range) of emicizumab. This property confers a competitive advantage to FVIII in assembling the intrinsic tenase complex and promoting FXa generation. Furthermore, FVIIIa activity is strictly regulated by natural anticoagulants, such as activated protein C and antithrombin, which help to limit thrombin generation and reduce the risk of hypercoagulability.

However, in patients with inhibitors, the concomitant use of emicizumab and BPAs still presents challenges regarding both safety and hemostatic efficacy. Concerns persist about the potential of thrombotic complications, especially with aPCC, as well as the variability in clinical response observed with rFVIIa.

In agreement with previous studies [15,32], our results also demonstrated that the in vitro addition of aPCC to plasma samples containing emicizumab can markedly increase thrombin generation indicating a strong synergistic effect. This effect is likely due to aPCC’s high content of prothrombin (substrate for thrombin generation) and procoagulant factors such as FIX/FIXa and FX/FXa, which facilitate the formation of emicizumab-mediated tenase complexes. However, the occurrence of this synergistic effect in vivo remains uncertain. Indeed, Kizilocak et al. [32] recently reported that emicizumab-treated patients who received therapeutic doses of aPCC did not exhibit excessive thrombin generation in post-infusion plasma samples, in contrast with the marked increases observed in vitro using aPCC-spiked plasma from the same patients. This discrepancy may be attributed to the fact that within the circulatory system, FIXa derived from aPCC can be inhibited by antithrombin or rapidly cleared from the bloodstream by cells expressing receptors such as the low-density lipoprotein receptor-related protein 1 (LRP1) [33].

In accordance to earlier findings [12,29,34], we demonstrated that the administration of rFVIIa in combination with emicizumab, the clinically recommended BPA, normalizes coagulation parameters without inducing excessive thrombin generation. This combination is considered safe, with no thrombotic events reported in the clinical context of its use. However, the short half-life of rFVIIa, its variable efficacy, lack of standardized monitoring assays, and high treatment cost remain significant limitations. Moreover, recent findings suggest that intensive rFVIIa use during emicizumab prophylaxis may impair its efficacy due to factor X consumption [13,35]. These findings underscore the need for optimized dosing protocols and alternative therapeutic strategies.

Our study revealed that rFIX concentrates are effective procoagulant agents in emicizumab-treated SHA patients, offering a promising alternative to conventional BPAs. All rFIX products tested—rFIX-alfa, rFIX-gamma, and rFIX-FP—produced a dose-dependent enhancement of thrombin generation and clot formation, reaching levels comparable to those achieved with rFVIIa or rFVIII ex vivo in CAT and ROTEM^®^ assays.

rFIX-alfa demonstrated a stronger procoagulant effect at lower doses, attributable to its higher content of activated FIXa compared to rFIX-gamma or rFIX-FP (6–10 times more). After blocking of FIXa using EGRck, the differences in procoagulant effect among products were abolished.

The presence of FIXa in FIX concentrates poses both a therapeutic opportunity and a potential safety concern. While FIXa enhances efficacy, it may also increase the risk of thrombotic events, particularly in combination with emicizumab. Thus, selecting products with lower FIXa content, such as nonacog gamma, or EHL formulations like rFIX-FP, may offer a safer profile and may provide a controlled and effective hemostatic response in emicizumab-treated patients.

Compared to traditional BPAs, rFIX concentrates offer important clinical advantages: a more physiologic mode of action via the intrinsic tenase complex, predictable dose–response, routine monitoring with standard FIX assays, reduced immunogenicity, and potentially lower thrombotic risk. EHL products also provide sustained coverage with less frequent dosing.

Our findings also suggest that the presence of FVIII inhibitors may enhance the FIX-dependent hemostatic efficacy of emicizumab. Thrombin generation assays revealed a markedly greater response to rFIX spiking in samples from patients with FVIII inhibitors, whereas no significant difference was observed with rFVIIa (Figure 2), which promotes coagulation by directly activating both FIX and FX. This highlights FIX concentration as a rate-limiting factor in emicizumab-mediated coagulation. Notably, while baseline levels of emicizumab, FIX, and FX did not differ significantly between patients with or without inhibitors (Table 1), the enhanced thrombin generation in the inhibitor group points toward a functional modulation of FIX availability.

To further investigate this phenomenon, we conducted spiking experiments with neutralizing anti-FVIII antibodies in plasma from patients without a prior history of inhibitors. Remarkably, the addition of these antibodies reproduced the enhanced thrombin generation seen in inhibitor-positive patients. This indicates that FVIII inhibition alone may be sufficient to potentiate emicizumab activity. These results support the hypothesis that residual or mutated endogenous FVIII may compete with emicizumab for binding to FIXa and FX. The presence of FVIII inhibitors would relieve this competition, thereby enhancing emicizumab’s efficacy upon the addition of rFIX. Although this competition model is the most plausible explanation, the complexity of the in vivo environment requires further consideration. For example, it could be hypothesized that a chronic, low-grade coagulation activation state, sometimes associated with high-titer inhibitors, could contribute to a ‘primed’ hemostatic system that responds more vigorously to a FIX trigger.

Additionally, to determine whether specific *F8* mutations, rather than the presence of inhibitors, could be responsible for the observed differences in hemostatic response, the patients’ molecular biology was also evaluated (Supplementary Table S2). In our cohort, the prevalence of Int22Inv (41%) is consistent with the reported data for SHA. By analyzing a subgroup of patients with the same Int22Inv mutation, we aimed to isolate the effect of inhibitors from the mutational background. The finding that thrombin peaks remained significantly higher in the inhibitor group (Supplementary Figure S2), even when the genetic variant was identical, suggests that the enhanced procoagulant response to rFIX in these patients is not mainly caused by differences in the underlying mutation.

These findings are consistent with the effective use of emicizumab in acquired HA [36,37], where FVIII autoantibodies are present, as well as in hemophilia B patients harboring FIX mutations that impair FVIII interaction [38]. In both scenarios, emicizumab has been shown to restore thrombin generation. Together, our data provide mechanistic insights into the interaction between emicizumab, FIX availability, and FVIII inhibition, with important implications for optimizing emicizumab-based therapy—not only in SHA patients with FVIII inhibitors, but potentially in selected cases of hemophilia B and acquired HA.

The present study has some limitations. First, the sample size, particularly in subgroup analyses, was limited. Second, the findings are based on ex vivo models, which may not fully recapitulate the complexity of in vivo hemostatic responses because they fail to capture interactions with the vascular endothelium, clearance pathways, and other physiological modulators of coagulation. Nevertheless, while these are ex vivo spiking experiments, preliminary in vivo data obtained recently by our group using a hemophilia A mouse model [39] corroborate these findings. Initial results from this model suggest that co-administering rFIX (100–300 U/kg) and emicizumab can restore hemostasias to levels comparable with those observed in wild-type mice. No immediate safety concerns were identified in the tested conditions. However, the potential for increased thrombotic risk remains a critical consideration, especially given that emicizumab already provides a steady procoagulant base. Therefore, a key focus of future research will be to extend these findings to thrombosis-specific models. Such studies will be crucial in accurately defining the in vivo safety margin and the real thrombotic potential of increasing FIX levels during emicizumab therapy.

## 5. Conclusions

Our data show that emicizumab-treated SHA patients, despite prophylaxis, maintain a suboptimal coagulation profile that can be sensitively evaluated using ROTEM^®^ and CAT under specific conditions. While rFVIIa and FVIII remain effective in restoring hemostasis, FIX concentrates also exhibit significant in vitro procoagulant potential, especially in patients with inhibitors. These findings suggest that FIX concentrates—particularly formulations with low FIXa content—could potentially serve as alternatives to BPAs in selected clinical scenarios, particularly in patients with FVIII inhibitors. These innovative results may open new horizons for the clinical practice in hemophilia, but they must be confirmed through in vivo studies to evaluate the clinical safety and efficacy of the concomitant use of FIX and emicizumab.

## Figures and Tables

**Table 1:** Characteristics of patients with severe hemophilia A (SHA) on prophylaxis with emicizumab, without (SHA_woInh) and with anti-FVIII inhibitors (SHA_Inh).

Variable	Reference Range	SHA Overall *n* = 20	SHA_WoInh *n* = 12	SHA_Inh *n* = 8	*p*-Value
Age, y	n/a	45 (27–54)	52 (44–54)	24 (17–39)	<0.02 *
Platelets, ×10^3^/µL	[150–370]	225 (180–251)	225 (180–250)	226 (181–258)	>0.8
Erythrocytes, ×10^6^/µL	[4–6]	5.04 (4.8–5.5)	4.9 (4.8–5.4)	5.4 (5.1–5.6)	<0.05 *
Fibrinogen, mg/dL	[150–450]	302 (258–323)	310 (276–353)	265 (215–318)	>0.09
PT-INR	[0.8–1.2]	1 (1.0–1.0)	1 (1.0–1.1)	1 (1.0–1.0)	>0.9
aPTT ratio	[0.8–1.2]	0.77 (0.75–0.82)	0.79 (0.75–0.85)	0.75 (0.71–0.80)	>0.3
Emicizumab, µg/mL	n/a	47.9 (37.3–62.5)	45.7 (33.0–57.7)	64.9 (38.6–85.1)	>0.06
FVIII inhibitor, BU/mL	n/a	n/a	n/a	1.4 (1.0–30.1)	n/a
FIX:C, IU/dL	[50–150]	111 (104–123)	118 (109–127)	100 (90–113)	>0.1
FX:C, IU/dL	[50–150]	126 (116–137)	130 (107–140)	123 (120–128)	>0.5
ABR before Emi.	n/a	6 (2.3–11.8)	7.5 (2.5–30)	5.5 (1.5–6)	>0.2
ABR after Emi.	n/a	0 (0–1)	0.5 (0–1)	0 (0–1)	>0.4

Abbreviations: PT-INR, prothrombin time -international normalized ratio; SHA severe hemophilia A; SHA patients with anti-FVIII inhibitors (>1 BU/mL) (SHA_Inh), SHA patients without inhibitors (SHA_woInh); aPTT, activated partial thromboplastin time; aPTT ratio is a calculation which compares the patient’s APTT value against the normal clotting time value. aPTT test is affected by emicizumab. FIX:C, activity of FIX by chromogenic assay (CSA); FX:C, clotting activity of FX by 1-stage PT-based assay (OSA). n/a: not applicable. Results are expressed as median (IQR). Reference range: [indicated between brackets]. *p* value was determined between SHA patients without (SHA_woInh) and with anti-FVIII inhibitors (SHA_Inh) using the Student t or Mann–Whitney *U* test; significance at *p* < 0.05. * *p* < 0.05.

**Table 2:** FIX activity assay of the rFIX concentrates used in the study.

rFIX Concentrate	OD Units	% FIXa
hFIXa (10 IU)	1.2	100
rFIX-alfa (10 IU)	0.076	6.3
rFIX-gamma (10 IU)	0.018	1.5
rFIX-FP (10 IU)	0.012	1.0
rFIX-alfa-EGRck blocked (10 IU)	0.007	0.6
rFIX-gamma-EGRck blocked (10 IU)	0.000	0.0
rFIX-FP -EGRck blocked (10 IU)	0.000	0.0

Levels of activated Factor IX (FIXa) in rFIX concentrates were quantified by adding the Spectrozyme^®^ FIXa chromogenic substrate and measuring the optical density (OD) at 405 nm. OD units: Absorbance values (OD_405_) obtained in 10 IU of rFIX concentrates using the Spectrozyme^®^ FIXa assay. Percentage (%) of FIXa was obtained using 10 IU of purified, activated human hFIXa as the 100% reference in the same assay.

## Data Availability

The original contributions presented in this study are included in the article and Supplementary Materials. Further inquiries can be directed to the corresponding authors.

## Supplementary Materials

The following supporting information can be downloaded at: https://www.mdpi.com/article/10.3390/biomedicines14040777/s1, Table S1: Clinical characteristics of patients with severe hemophilia A included in the study; Table S2: Genetic variants identified in the F8 gene of patients with severe hemophilia A included in the study; Tables S3: Results of statistical analyses of data shown in Figure 1. Differences in CT or MAXVt of ROTEM in all experimental conditions between patients without and with inhibitors; Tables S4: Results of statistical analyses of data shown in Figure 2. Differences in peak or ETP of thrombin generation in all experimental conditions between patients without and with inhibitors; Tables S5: Results of statistical analyses of data shown in Figure 3A. Differences in CT between all the experimental conditions (basal and each concentration of FVIII, rFVII, rFIX-alfa, rFIX-gamma, and rFIX-FP); Tables S6: Results of statistical analyses of data shown in Figure 3B. Differences in peak between all the experimental conditions (basal and each concentration of FVIII, rFVII, rFIX-alfa, rFIX-gamma, and rFIX-FP); Tables S7: Results of statistical analyses of data shown in Figure 4. Differences in ROTEM’s CT or CAT’s peak of different FIX, untreated or EGRck-blocked. Figure S1: Evaluation of clot formation in blood samples from emicizumab-treated SHA patients using two different ROTEM^®^ tests; Figure S2: Comparison of the procoagulant effects of rFIX concentrates between emicizumab-treated SHA patients with Intron-22 inversion, with and without inhibitors. Supplementary Methods: Development of FVIII inhibitors in hemophilia A mice.
