# Supplementary material for: Procoagulant Effect of FIX Concentrates and Bypass Agents in Combination with Emicizumab and Impact of FVIII Inhibitors
Source: Biomedicines. 2026 Mar 29;14(4):777. doi: 10.3390/biomedicines14040777 (PMC13113436; doi:10.3390/biomedicines14040777)
Supplement: Supplementary file 1 [file biomedicines-14-00777-s001.zip › biomedicines-4179369-supplementary.pdf]

Supplementary Materials:

Title: Procoagulant effect of FIX concentrates and bypass agents in combination with emicizumab and impact of FVIII inhibitors

Authors: Elena G. Arias-Salgado[1#], María Teresa Álvarez Román[1,2], Abel Dos Santos Ortas[1], Ihosvany Fernandéz-Bello[1], Elena Monzón Manzano[1], Paula Acuña[1], Mónica Martín Salces[1], Maria Isabel Rivas Pollmar[1], Sara García Barcenilla[1], Nora V. Butta[1], Víctor Jimenéz-Yuste[1,2#]

Supplementary Table S1: Clinical characteristics of patients with severe Hemophilia A included in the study

Patient_ID	Inhibitor (BU)	Age (years)	Platelet count ($x10^3/\mu l$) [150-370]	Erithrocyte count ($x10^6/\mu l$) [4.3-5.75]	Fibrinogen Derived (mg/dl) [150-450]	Fibrinogen Clauss (mg/dl) [150-450]	FIX:C CSA (IU/dl) [50-150]	FX:C OSA (IU/dl) [50-150]	PT-INR [0.8-1.2]	aPTT ratio [0.8-1.2]	Emicizumab (µg/ml)	Dose of Emicizumab prophylaxis	Concomitant medications	Annual Bleeding Rate (ABR) before Emicizumab	Annual Bleeding Rate (ABR)	Annual Joint Bleeding Rate (AJBR)
**E01**	3.7	16	168	4.81	254	ND	88.6	122.1	1	0.75	62.8	1.5mg/kg/week	Methylphenidate / guanfacina	12	1	0
**E02**	1.1	17	285	5.58	210	285	97.4	119.4	1.1	0.8	40	6mg/kg/every 4 weeks	No	5	0	0
**E03**	1	24	215	5.69	211	298	110.5	113.1	1	0.85	37	1.5mg/kg/week	No	6	0	0
**E04**	no	53	220	4.76	264	315	121.5	124.9	1	0.75	46	6mg/kg/every 4 weeks	rilpivirina (25mg/day), dolutegravir (50mg/day), irbesartan (300mg/day), amlodipine 5 (1/day). metformin (850mg/day), celecoxib (demand)	30	5	4
**E05**	no	47	251	4.71	299	318	153.4	127.9	1.1	0.79	28.9	6mg/kg/every 4 weeks	etravirine (400mg/day), raltegravir (800mg/day), metformin (850mg/day), pantoprazol (40mg/day), Famotidine (40mg/day), irbersartan (150mg/day), amlodipine (5mg/day), spironolactone (50mg/day), allopurinol (100mg/day), calciferol (1/week), levothyroxine (50mg/day), celecoxib (200mg/demand)	5	1	1
**E06**	no	54	177	4.97	316	377	104.4	102.9	1.1	0.91	14.4	6mg/kg/every 4 weeks	zolpidem(10mg/day), lercanidipine 20/10mg. carvedilol (25mg/day); hidroferol (1/15days), allopurinol (100mg/day), rosuvastatina (5mg/day), prednisone (5-2.5/48h), methotrexate(12.5mg/week), folic acid (1/week), etoricoxib (demand), tramadol (demand)	4	1	1
**E07**	no	41	199	5.68	322	368	116	132.4	0.9	0.83	57.4	3mg/kg/every 2 weeks	dolutegravir/rilpivirine (25/50mg/day), levothyroxine (50mg/day), rosuvastatin (5mg/day), calciferol (1/month)	11	0	0
**E08**	no	54	277	4.86	516	445	109	105	1	0.78	36.4	1.5mg/kg/week	emtricitabine/tenofovir (200/300mg/day), raltegravir (400mg/12h), calciferol (0,266mg/month), metformin (850mg/day)	49	0	0
**E09**	no	50	138	4.8	303	311	136.2	145.8	1	0.71	57.8	3mg/kg/every 2 weeks	lamivudine (300 mg/day), dolutegravir (50mg/day), etoricoxib (demand), zolpidem (demand)	90	0	0
**E10**	no	44	190	4.9	256	260	108.7	101.8	1.1	0.75	45.3	1.5mg/kg/week	celecoxib (200mg/demand) Frenadol (demand) emtricitabine/tenofovir (200/300mg/day) raltegravir (400mg/12h)	30	0	0
**E11**	no	45	266	5.51	381	324	127.9	140.6	1	0.85	31.9	1.5mg/kg/week	darunavir/cobicistat (800/150mg/day), atorvastatina (10 mg/day), omeprazole (20 mg/day)	9	1	0
**E12**	no	53	246	5.48	363	426	111.2	132.4	1	0.8	44.2	6mg/kg/every 4 weeks	enalapril (20 mg/day)	1	2	2
**E13**	168	41	261	5.24	367	354	113.7	142.3	1	0.81	38.1	6mg/kg/every 4 weeks	No	6	1	0
**E14**	no	54	230	4.79	311	359	122.9	114.3	1	0.9	49.8	6mg/kg/every 4 weeks	dolutegravir /rilpivirine (50/25mg/day), irbersartan/hidroclorotiazida (150/12.5 mg/day), calcifediol (0.266mg/15days), pitavastina (2mg/day)	6	0	0
**E15**	no	54	173	4.86	309	301	107.1	138.9	1	0.76	58.9	1.5mg/kg/week	emtricitabina/rilpivirina/ tenofovir (200/25/25mg/day), carteolol (20mg/ml collyrium)	0	0	0
**E17**	no	34	237	4.52	268	286	120.5	161.5	0.9	0.65	61.7	1.5mg/kg/week	tramadol (100mg/3times/week), metamizol (demand), celecoxib (demand), etoricoxib (demand)	2	1	1
**E18**	38.9	34	176	5.38	323	275	103.3	122.1	1	0.7	85.3	1.5mg/kg/week	celecoxib (demand), paracetamol (demand)	6	0	0
**E19**	1	65	249	5.52	275	323	153.2	123.5	1	0.75	66.9	1.5mg/kg/week	enalpril (20mg/day)	0	0	0
**E20**	1.7	23	194	5.37	300	316	57.7	127.9	1	0.74	89.2	1.5mg/kg/week	No	3	1	1
**E21**	1.1	18	237	5.1	226	220	95	127.9	1	0.7	84.4	1.5mg/kg/week	No	1	0	0

Supplementary Table S2: Genetic variants identified in the F8 gene of patients with severe Hemophilia A included in the study

Patient_ID	Variant (HGVS)	Protein_effect	Variant_type
**E1**	NA	NA	NA
**E2**	F8(NM_000132.4):c.1752+2T>A	p.?	Splice-site
**E3**	F8 intron 22 inversion	—	Inversion
**E4**	F8(NM_000132.4):c.2373G>A	p.(Trp791Ter)	Nonsense
**E5**	F8(NM_000132.4):c.5953C>T	p.(Arg1985Ter)	Nonsense
**E6**	F8 intron 22 inversion	—	Inversion
**E7**	F8 intron 22 inversion	—	Inversion
**E8**	NA	NA	NA
**E9**	F8(NM_000132.4):c.6211A>G	p.(Arg2071Gly)	Missense
**E10**	F8(NM_000132.4):c.3637del	p.(Ile1213PhefsTer5)	Frameshift
**E11**	F8(NM_000132.4):c.6403C>T	p.(Arg2135Ter)	Nonsense
**E12**	F8 intron 22 inversion	—	Inversion
**E13**	F8 deletion exons 11–25	—	Large deletion
**E14**	F8 intron 22 inversion	—	Inversion
**E15**	F8 intron 22 inversion	—	Inversion
**E17**	F8 intron 22 inversion	—	Inversion
**E18**	F8(NM_000132.4):c.5274del	p.(Asp1759MetfsTer13)	Frameshift
**E19**	NA	NA	NA
**E20**	F8 intron 22 inversion	—	Inversion
**E21**	F8(NM_000132.4):c.1063C>T	p.(Arg355Ter)	Nonsense

NA – *Not Available*

Supplementary Table S3:

Table S3 shows the differences in CT or MAXVt of ROTEM in all experimental conditions between patients without (woInh) and with inhibitors (Inh). Depending on whether the data distributions were normal, a Student's t-test or a Mann–Whitney U test was performed. A p-value of less than 0.05 is considered statistically significant.

CT (s)	woInh (mean±SD)	Inh (mean±SD)	p	MAXVt (s)	woInh (mean±SD)	Inh (mean±SD)	p
**Basal**	2744±853	2168±459	0.099	**Basal**	3507±318	2956±301	0.279
**0.01 U/ml aPCC**	724±270	664±96	0.613	**0.01 U/ml aPCC**	1134±118	1239±126	0.670
**0.05 U/ml aPCC**	405±127	389±94	0.778	**0.05 U/ml aPCC**	597±44	505±132	0.408
**0.5 U/ml aPCC**	207±51	217±28	0.651	**0.5 U/ml aPCC**	285±17	285±11	0.994
**0.05 µg/ml rFVIIa**	902±285	848±397	0.771	**0.05 µg/ml rFVIIa**	1542±224	1232±246	0.443
**0.25 µg/ml rFVIIa**	764±279	741±305	0.866	**0.25 µg/ml rFVIIa**	1064±122	1091±183	0.901
**1 µg/ml rFVIIa**	651±205	657±226	0.953	**1 µg/ml rFVIIa**	867±86	964±165	0.578

Supplementary Table S4:

Table S4 shows the differences in peak or ETP of thrombin generation in all experimental conditions between patients without (woInh) and with inhibitors (Inh). Depending on whether the data distributions were normal, a Student's t-test or a Mann–Whitney U test was performed. A p-value of less than 0.05 is considered statistically significant.

**Peak (nM)**	**woInh** (mean±SD)	**Inh** (mean±SD)	**p**	**ETP (nM.min)**	**woInh** (mean±SD)	**Inh** (mean±SD)	**p**
**Basal**	17±3	24±4	0.168	**Basal**	337±53	539±80	0.042
**0.01 U/ml aPCC**	49±6	55±5	0.544	**0.01 U/ml aPCC**	751±74	815±86	0.593
**0.05 U/ml aPCC**	135±11	148±6	0.412	**0.05 U/ml aPCC**	1321±92	1371±68	0.701
**0.5 U/ml aPCC**	452±21	462±23	0.770	**0.5 U/ml aPCC**	3245±189	3521±386	0.494
**0.05 µg/ml rFVIIa**	32±4	37±5 NS	0.513	**0.05 µg/ml rFVIIa**	580±67	648±110	0.584
**0.25 µg/ml rFVIIa**	43±5	50±6	0.503	**0.25 µg/ml rFVIIa**	707±67	876±83	0.205
**1 µg/ml rFVIIa**	66±7	72±8	0.601	**1 µg/ml rFVIIa**	910±83	1011±105	0.470

Supplementary Table S5:

Table S5 shows the differences between all the experimental conditions (basal and each concentration of FVIII, rFVII, rFIX-alfa, rFIX-gamma, and rFIX-FP). A one-way ANOVA followed by a post-hoc Tukey or a Kruskal–Wallis test followed by a post-hoc Dunn test was performed, depending on whether the data distributions were normal. A p-value of less than 0.05 is considered statistically significant.

[Reagent]= 1	CT (s)	p	[Reagent]=0.5	CT (s)	p	[Reagent]= 1.5	CT (s)	p
**Basal**	2572±754	****	**Basal**	2572±754	ns	**Basal**	2572±754	****
**rFVIIa** (1 µg/ml)	632±214		**rFVIII** (0.5 IUml)	1643±537		**rFIX-gamma** (1.5 IUml)	851±255	
**Basal**	2572±754	ns	**Basal**	2572±754	****	**Basal**	2572±754	****
**rFVIII** (1 IUml)	1480±504		**rFIX-alfa** (0.5 IUml)	655±177		**rFIX-FP** (1.5 IUml)	988±375	
**Basal**	2572±754	****	**Basal**	2572±754	*	**rFIX-gamma** (1.5 IUml)	1233±522	ns
**rFIX-alfa** (1 IUml)	514±116		**rFIX-gamma** (0.5 IUml)	1233±522		**rFIX-FP** (1.5 IUml)	988±375	
**Basal**	2572±754	***	**Basal**	2572±754	ns			
**rFIX-gamma** (1 IUml)	943±319		**rFIX-FP** (0.5 IUml)	1523±598				
**Basal**	2572±754	ns	**rFVIII** (0.5 IUml)	1643±537	*			
**rFIX-FP** (1 IUml)	1224±497		**rFIX-alfa** (0.5 IUml)	655±177				
**rFVIIa** (1 µg/ml)	632±214	**	**rFVIII** (0.5 IUml)	1643±537	ns			
**rFVIII** (1 IUml)	1480±504		**rFIX-gamma** (0.5 IUml)	1233±522				
**rFVIIa** (1 µg/ml)	632±214	ns	**rFVIII** (0.5 IUml)	1643±537	ns			
**rFIX-alfa** (1 IUml)	514±116		**rFIX-FP** (0.5 IUml)	1523±598				
**rFVIIa** (1 µg/ml)	632±214	**	**rFIX-alfa** (0.5 IUml)	655±177	ns			
**rFIX-FP** (1 IUml)	1224±497		**rFIX-gamma** (0.5 IUml)	1233±522				
**rFVIIa** (1 µg/ml)	632±214	ns	**rFIX-alfa** (0.5 IUml)	655±177	*			
**rFIX-gamma** (1 IUml)	943±319		**rFIX-FP** (0.5 IUml)	1523±598				
**rFVIII** (1 IUml)	1480±504	***	**rFIX-FP** (0.5 IUml)	1523±598	ns			
**rFIX-alfa** (1 IUml)	514±116		**rFIX-gamma** (0.5 IUml)	1233±522				
**rFVIII** (1 IUml)	1480±504	ns						
**rFIX-gamma** (1 IUml)	943±319							
**rFVIII** (1 IUml)	1480±504	ns						
**rFIX-FP** (1 IUml)	1224±497							
**rFIX-alfa** (1 IUml)	514±116	*						
**rFIX-gamma** (1 IUml)	943±319							
**rFIX-alfa** (1 IUml)	514±116	***						
**rFIX-FP** (1 IUml)	1224±497							
**rFIX-gamma** (1 IUml)	943±319	ns						
**rFIX-FP** (1 IUml)	1224±497							

Supplementary Table S6:

Table S6 shows the differences between all the experimental conditions (basal and each concentration of FVIII, rFVII, rFIX-alfa, rFIX-gamma, and rFIX-FP). A one-way ANOVA followed by a post-hoc Tukey or a Kruskal–Wallis test followed by a post-hoc Dunn test was performed, depending on whether the data distributions were normal. A p-value of less than 0.05 is considered statistically significant.

[Reagent]= 1	Peak (nM)	p
**Basal**	20±12	****
**rFVIIa** (1 µg/ml)	68±24	
**Basal**	20±12	****
**rFVIII** (1 IUml)	75±24	
**Basal**	20±12	****
**rFIX-alfa** (1 IUml)	122±37	
**Basal**	20±12	*
**rFIX-gamma** (1 IUml)	49±21	
**Basal**	20±12	ns
**rFIX-FP** (1 IUml)	44±29	
**rFVIIa** (1 µg/ml)	68±24	ns
**rFVIII** (1 IUml)	75±24	
**rFVIIa** (1 µg/ml)	68±24	****
**rFIX-alfa** (1 IUml)	122±37	
**rFVIIa** (1 µg/ml)	68±24	ns
**rFIX-FP** (1 IUml)	44±29	
**rFVIIa** (1 µg/ml)	68±24	ns
**rFIX-gamma** (1 IUml)	49±21	
**rFVIII** (1 IUml)	75±24	***
**rFIX-alfa** (1 IUml)	122±37	
**rFVIII** (1 IUml)	75±24	ns
**rFIX-gamma** (1 IUml)	49±21	
**rFVIII** (1 IUml)	75±24	ns
**rFIX-FP** (1 IUml)	44±29	
**rFIX-alfa** (1 IUml)	122±37	****
**rFIX-gamma** (1 IUml)	49±21	
**rFIX-alfa** (1 IUml)	122±37	****
**rFIX-FP** (1 IUml)	44±29	
**rFIX-gamma** (1 IUml)	49±21	ns
**rFIX-FP** (1 IUml)	44±29	

[Reagent]= 0.5	Peak (nM)	p
**Basal**	20±12	**
**rFVIII** (0.5 IUml)	48±26	
**Basal**	20±12	****
**rFIX-alfa** (0.5 IUml)	79±25	
**Basal**	20±12	ns
**rFIX-gamma** (0.5 IUml)	36±17	
**Basal**	20±12	ns
**rFIX-FP** (0.5 IUml)	31±18	
**rFVIII** (0.5 IUml)	48±26	**
**rFIX-alfa** (0.5 IUml)	79±25	
**rFVIII** (0.5 IUml)	48±26	ns
**rFIX-gamma** (0.5 IUml)	36±17	
**rFVIII** (0.5 IUml)	48±26	ns
**rFIX-FP** (0.5 IUml)	31±18	
**rFIX-alfa** (0.5 IUml)	79±25	****
**rFIX-gamma** (0.5 IUml)	36±17	
**rFIX-alfa** (0.5 IUml)	79±25	****
**rFIX-FP** (0.5 IUml)	31±18	
**rFIX-FP** (0.5 IUml)	31±18	ns
**rFIX-gamma** (0.5 IUml)	36±17	

[Reagent]= 1.5	Peak (nM)	p
**Basal**	20±12	****
**rFIX-gamma** (1.5 IUml)	61±26	
**Basal**	20±12	***
**rFIX-FP** (1.5 IUml)	51±30	
**rFIX-gamma** (1.5 IUml)	61±26	ns
**rFIX-FP** (1.5 IUml)	51±30	

Supplementary Table S7:

Table S7 shows the differences in ROTEM´s CT or CAT´s Peak of different FIX, untreated or EGRck-blocked. Depending on whether the data distributions were normal, a Student’s t-test or a Mann–Whitney U test was performed. A p-value of less than 0.05 is considered statistically significant.

**CT (s)**	**Untreated** (mean±SD)	**EGRck-blocked** (mean±SD)	**p**	**Peak (nM)**	**Untreated** (mean±SD)	**EGRck-blocked** (mean±SD)	**p**
**Basal**	2524±1111			**Basal**	20±10		
**0.5 IU/ml rFIX-alfa**	727±199	2393±476	0.029	**1 IU/ml rFIX-alfa**	114±20	30±8	0.029
**1.5 IU/ml rFIX-gamma**	821±238	1924±235	0.028	**1.5 IU/ml rFIX-gamma**	58±10	27±7	0.029
**1.5 IU/ml rFIX-FP**	967±219	1608±215	0.029	**1.5 IU/ml rFIX-FP**	43±11	38±9	0.65

Supplementary Figure S1:

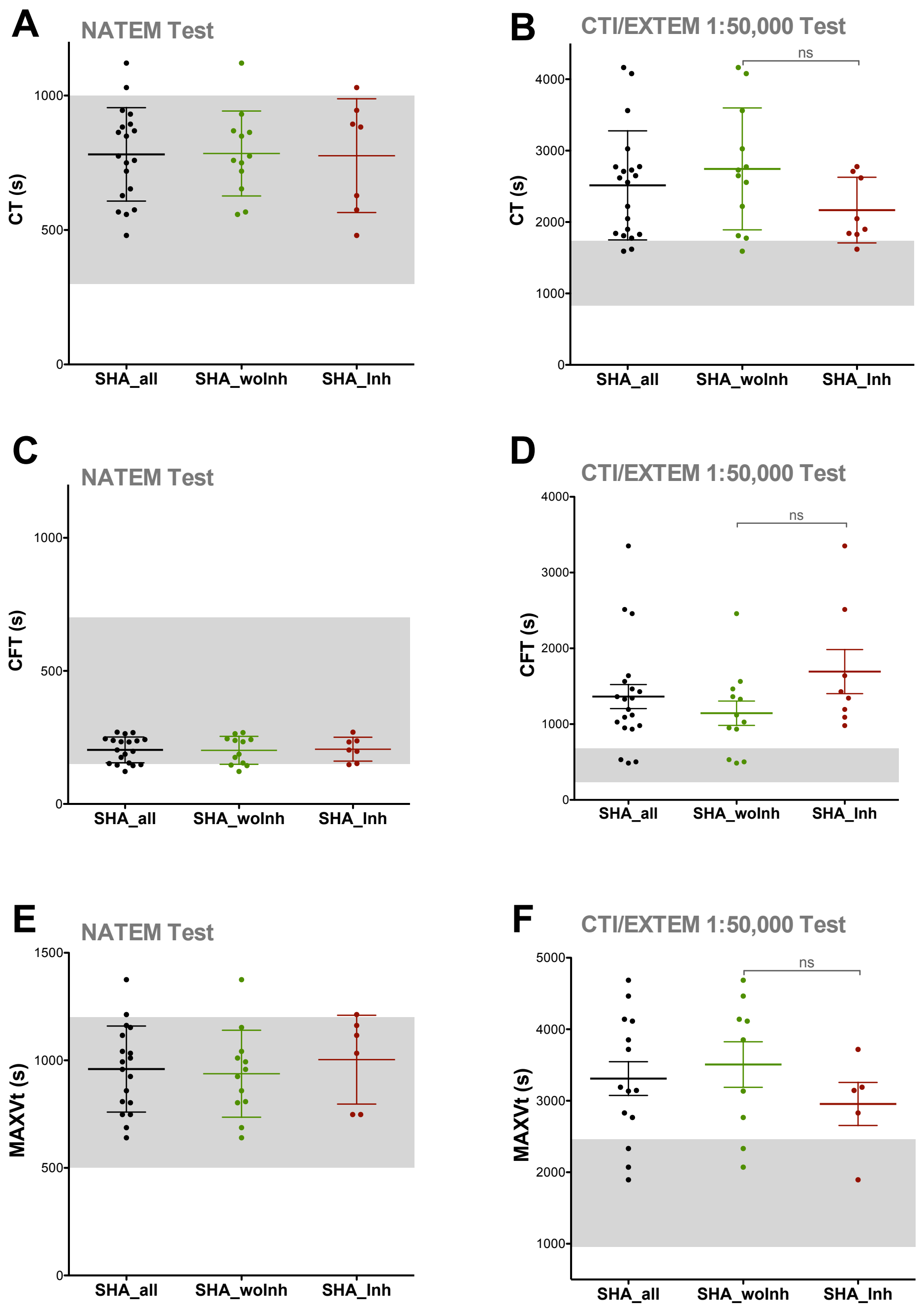

Supplementary Figure S1: Evaluation of clot formation in blood samples from emicizumab-treated SHA patients using two different ROTEM® tests. CT (**A, B**), CFT (**C, D**), and MAXVt (**E, F**) obtained by ROTEM® using NATEM test in citrated blood sample (**A, C, E**) or using EXTEM reagent diluted 1:50,000 in blood samples collected with CTI (**B, D, F**) of emicizumab-treated SHA patients. Data are shown for all patients (SHA_all), with (SHA_Inh) and without inhibitor (SHA_woInh). Bars represent mean ± SD. Grey-shaded areas correspond to the reference range obtained from healthy controls. *ns*: non-significant difference (p>0.05).

Supplementary Figure S2:

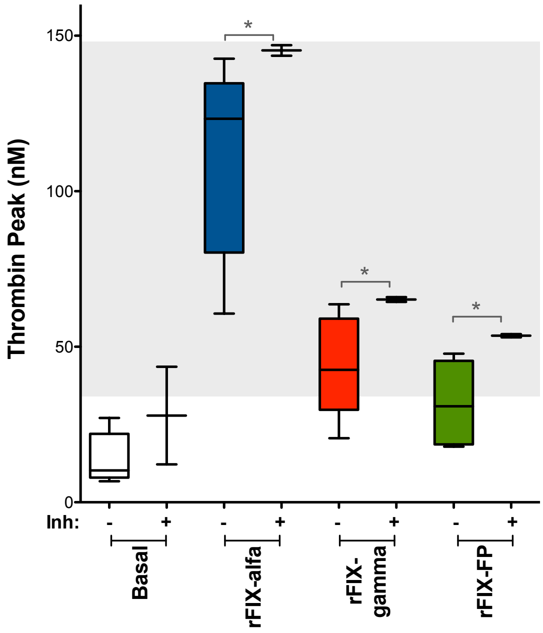

Supplementary Figure S2: Comparison of the procoagulant effects of rFIX concentrates between emicizumab-treated SHA patients with Intron-22 Inversion, with and without inhibitors. Thrombin peak obtained by CAT assays after *the ex vivo* addition of rFIX concentrates (rFIX-alfa, rFIX-gamma, and rFIX-FP) (1 IU/mL) to CTI samples from emicizumab-treated SHA patient with Intron-22 Inversion, with (+) or without inhibitors (-). Box plots showing the 90/10 percentile at the whiskers and the median by line. Grey-shaded areas correspond to the reference range obtained from healthy controls. Statistical differences between groups were assessed using Welch's t-test**.** *p<0.05.

Supplementary Methods

Development of FVIII inhibitors in hemophilia A mice

1. Study design

Human factor VIII (FVIII) was administered to FVIII-deficient mice to induce the development of inhibitors against this coagulation factor. The presence and titers of FVIII inhibitors in mouse plasma were subsequently determined using the Bethesda assay, the standard method for quantifying inhibitors against FVIII.

For the experiments described in this study, plasma obtained from mice with confirmed FVIII inhibitors, as well as plasma from mice without detectable inhibitors, was used as an experimental reagent. Mouse plasma was added to patient plasma samples at a final dilution of 1:30 prior to thrombin generation analysis.

The number of animals used in this study was determined based on the volume of plasma required for the downstream experiments. Three mice per group were included, as this number provided sufficient plasma to perform thrombin generation assays in triplicate under all experimental conditions tested.

2. Animals and housing

A total of 6 mice of the B6;129S-F8tm1Kaz/J strain were used in this study (available from The Jackson Laboratory; https://www.jax.org/strain/004424) and obtained through the European distributor Charles River Laboratories. These mice are factor VIII (FVIII)-deficient knock-out animals. Only hemizygous male B6;129S-F8tm1Kaz/J mice, aged 8–10 weeks at the start of the study, were included.

Animals were housed in the authorized CIB-CSIC animal facility (Madrid, Spain) and were examined by trained animal facility personnel to assess health status and welfare. Mice were housed in cages appropriate for the species, with a minimum floor area of 330 cm^2, allowing housing of up to four mice weighing 25–30 g, in accordance with the minimum surface requirements established by spanish RD 53/2013. Environmental conditions were maintained at 20–22 °C, with 45–55% relative humidity and a 12 h light/12 h dark photoperiod. Animals had ad libitum access to food and water and were monitored daily.

Animals will be monitored daily by animal facility staff. The designated veterinarian and the Animal Welfare Officer will be consulted if any signs of illness, bleeding, or distress are observed. Animal posture, coat condition, activity, general health status, abnormal behaviours, and any signs of pain or stress will be evaluated. Due to the specific phenotype of these mice, particular attention will be paid to the presence of possible bleeding episodes, especially joint bleeding that may severely impair mobility or severe bleeding in soft tissues. All observations will be recorded using a monitoring sheet designed to assess these parameters.

The daily evaluation of each mouse will be documented using monitoring sheets that include a scoring system for different variables, as follows:

- Body weight loss: 0 points: ≤ 1%; 1 point: 10–15%; 2 points: ≥ 15%.
- Fecal consistency: 0 points: normal; 1 point: soft stools; 2 points: diarrhea.
- Presence of blood in feces: 0 points: negative; 1 point: occult blood; 2 points: visible blood.

- Appearance: 0 points: normal shiny coat; 1 point: poor coat condition and nasal secretions; 2 points: abnormal posture (hunched) and/or piloerection.
- Mobility: 0 points: normal; 1 point: reduced mobility; 2 points: inactivity.
- Bleeding: 2 points: bleeding in joints or soft tissues.

The criteria for applying humane endpoints, based on this clinical scoring system, will be the following:

- Any animal presenting a total score of 3–5 points in the daily assessment for two consecutive days.
- Any animal with a total score exceeding 6 points on any single day during the procedure.

3. Experimental procedures

a) Intravenous administration of human FVIII to induce inhibitor formation

To induce FVIII inhibitor development, three male B6;129S-F8tm1Kaz/J mice (8–10 weeks old) received repeated intravenous administrations of human FVIII. The protocol was adapted from previously published studies describing inhibitor development in this mouse model.

For the non-inhibitor control group, three age-matched male B6;129S-F8tm1Kaz/J mice received the same number of intravenous injections of saline solution.

Each mouse received six intravenous injections at two-week intervals. The procedure consisted of the following steps: placement of the mouse in a restraining device; tail warming by immersion in a 40 °C water bath for 5–10 minutes; and intravenous injection via the tail vein of 80 IU/kg human FVIII (hFVIII) (Kovaltry, Bayer), prepared at a concentration of 1 IU/20 µL. For a 25 g mouse, a dose of 2 IU FVIII in 80 µL was administered. Control mice received the same volume (80 µL) of saline solution.

Because these mice present coagulation defects, injections were performed using 30G or smaller needles (31G) to minimize bleedings. After injection, manual pressure was applied to the injection site for 1–2 minutes to ensure complete hemostasis. If necessary, a gelatin hemostatic sponge (Espongostan Film) was applied until bleeding stopped. All injections were performed at the same time of day to minimize circadian variability.

b) Blood collection

Previously treated B6;129S-F8tm1Kaz/J mice were anesthetized using inhaled isoflurane (see anesthesia section below). Blood collection followed a protocol optimized to preserve hemostatic parameters and minimize coagulation or hemolysis. The abdominal cavity was opened, and 180 µL of 3.2% sodium citrate was administered intravenously into the inferior vena cava (IVC) using a 23G needle and a 2 mL syringe, 15–20 seconds prior to total blood withdrawal. Complete blood collection was then performed using the same syringe.

c) Anesthesia

Inhalation anesthesia with isoflurane was used. Animals were placed in an anesthetic induction chamber with the vaporizer set to 5% isoflurane and an oxygen flow of 0.5–1.0 L/min until complete loss of consciousness.

Parenteral anesthetic agents were not administered in order to avoid potential interference with hemostatic or immune parameters evaluated in this study.

d) Euthanasia

Mice were anesthetized with isoflurane and euthanized by exsanguination. Death was confirmed using cervical dislocation and/or confirmation of rigor mortis.

4. Results: Determination of anti-hFVIII inhibitor titers

Plasma was prepared by centrifugation of blood samples (2500 × g, 10 min). Plasma samples were aliquoted and stored at −80 °C until analysis. Neutralizing anti-hFVIII titers were measured using the standard Nijmegen modification of the Bethesda assay. Descriptive statistics were used to report inhibitor titers. Results are presented as mean ± standard deviation (SD). Statistical analyses were performed using GraphPad Prism.

FVIII inhibitor activity was detected in all mice treated with hFVIII (n=3) (inhibitor titer: mean ± SE: 246 ± 63 BU; range: 31–258 BU). Control mice (n=3) receiving saline injections did not develop detectable FVIII inhibitors.
